# Universal subunit vaccine protects against multiple SARS-CoV-2 variants and SARS-CoV

**DOI:** 10.1038/s41541-024-00922-z

**Published:** 2024-07-25

**Authors:** Gang Wang, Abhishek K. Verma, Juan Shi, Xiaoqing Guan, David K. Meyerholz, Fan Bu, Wei Wen, Bin Liu, Fang Li, Stanley Perlman, Lanying Du

**Affiliations:** 1 Institute for Biomedical Sciences, Georgia State University, Atlanta, GA USA; 2 Department of Microbiology and Immunology, University of Iowa, Iowa City, IA USA; 3 Department of Pathology, University of Iowa, Iowa City, IA USA; 4 Department of Pharmacology, University of Minnesota Medical School, Minneapolis, MN USA; 5 Center for Coronavirus Research, University of Minnesota, Minneapolis, MN USA; 6 Hormel Institute, University of Minnesota, Austin, MN USA; 7 Department of Pediatrics, University of Iowa, Iowa City, IA USA

**Keywords:** SARS-CoV-2, SARS virus, Protein vaccines

## Abstract

Although Omicron RBD of SARS-CoV-2 accumulates many mutations, the backbone region (truncated RBD) of spike protein is highly conserved. Here, we designed several subunit vaccines by keeping the conserved spike backbone region of SARS-CoV-2 Omicron BA.1 subvariant (S-6P-no-RBD), or inserting the RBD of Delta variant (S-6P-Delta-RBD), Omicron (BA.5) variant (S-6P-BA5-RBD), or ancestral SARS-CoV-2 (S-6P-WT-RBD) to the above backbone construct, and evaluated their ability to induce immune responses and cross-protective efficacy against various SARS-CoV-2 variants and SARS-CoV. Among the four subunit vaccines, S-6P-Delta-RBD protein elicited broad and potent neutralizing antibodies against all SARS-CoV-2 variants tested, including Alpha, Beta, Gamma, and Delta variants, the BA.1, BA.2, BA.2.75, BA.4.6, and BA.5 Omicron subvariants, and the ancestral strain of SARS-CoV-2. This vaccine prevented infection and replication of SARS-CoV-2 Omicron, and completely protected immunized mice against lethal challenge with the SARS-CoV-2 Delta variant and SARS-CoV. Sera from S-6P-Delta-RBD-immunized mice protected naive mice against challenge with the Delta variant, with significantly reduced viral titers and without pathological effects. Protection correlated positively with the serum neutralizing antibody titer. Overall, the designed vaccine has potential for development as a universal COVID-19 vaccine and/or a pan-sarbecovirus subunit vaccine that will prevent current and future outbreaks caused by SARS-CoV-2 variants and SARS-related CoVs.

## Introduction

Severe acute respiratory syndrome coronavirus-2 (SARS-CoV-2) causes Coronavirus Disease 2019 (COVID-19), which was first emerged in 2019 and led to a global pandemic with devastating human deaths and economic losses; indeed, the virus remains a threat to public health worldwide^1,2^. SARS-CoV-2 has mutated frequently since its first emergence, with the most mutations being identified in the spike (S) protein, particularly the receptor-binding domain (RBD)^3,4^. These mutations have resulted in multiple variants of concern (VOCs), which include the previously circulating VOCs, such as Alpha, Beta, Gamma, Delta, and Omicron subvariants BA.1, BA.2 (BA.2.75), BA.3, BA.4 (BA.4.6), BA.5, XBB.1.5, and EG.5, as well as the currently circulating Omicron JN.1, KP.2, KP.3, and LB.1 subvariants^3^. Mutations in the S protein, particularly the RBD, of SARS-CoV-2, have led to a marked reduction in the efficacy of antibodies produced in response to the ancestral (wild-type (WT)) strain or early variants of SARS-CoV-2^5–8^. As such, broadly protective vaccines with potent neutralizing activity against multiple variants, including current and future SARS-CoV-2 VOCs with pandemic potential, are needed.

Another coronavirus, SARS-CoV, which caused a global outbreak during the 2002–2003, belongs to the same genus (beta-coronavirus) as SARS-CoV-2, and it uses the same cellular receptor (angiotensin converting enzyme-2 (ACE2)) for viral entry^9–11^. In addition, some SARS-related coronaviruses from bats also utilize the same ACE2 receptor to enter host cells^11,12^; these viruses have future pandemic potential. Recurrence of SARS-CoV-induced SARS and other diseases potentially caused by SARS-related CoVs highlights the importance and necessity of developing effective universal pan-coronavirus vaccines against not only SARS-CoV-2, but also SARS-CoV and other beta-coronaviruses with pandemic potential.

Among all SARS-CoV-2 and SARS-CoV proteins, the surface S protein and its RBD are critical targets for development of effective vaccines^9,13,14^. The S protein comprises two subunits: S1 and S2^15,16^. The RBD in the S1 subunit of SARS-CoV-2 Omicron VOCs has a significantly higher number of amino acid variations than the RBD of the earlier SARS-CoV-2 VOCs, including Delta; nevertheless, the backbone region (truncated RBD) of the S protein is highly conserved^17–19^.

Here, we designed several subunit vaccines based on the S protein backbone (without its own RBD) of SARS-CoV-2 Omicron BA.1 (S-6P) (which is relatively conserved among all SARS-CoV-2 strains and contains the HexaPro sequences, a C-terminal Foldon trimerization domain, and a His_6_ tag), with or without insertion of the respective RBD described below. We then evaluated their ability to induce broadly neutralizing antibodies and protection against multiple SARS-CoV-2 VOCs and SARS-CoV. Finally, we elucidated the mechanisms by which they induce protection against infection of SARS-CoV-2 and SARS-CoV.

## Results

### Characterization of the recombinant protein subunit vaccines

**Fig. 1:**
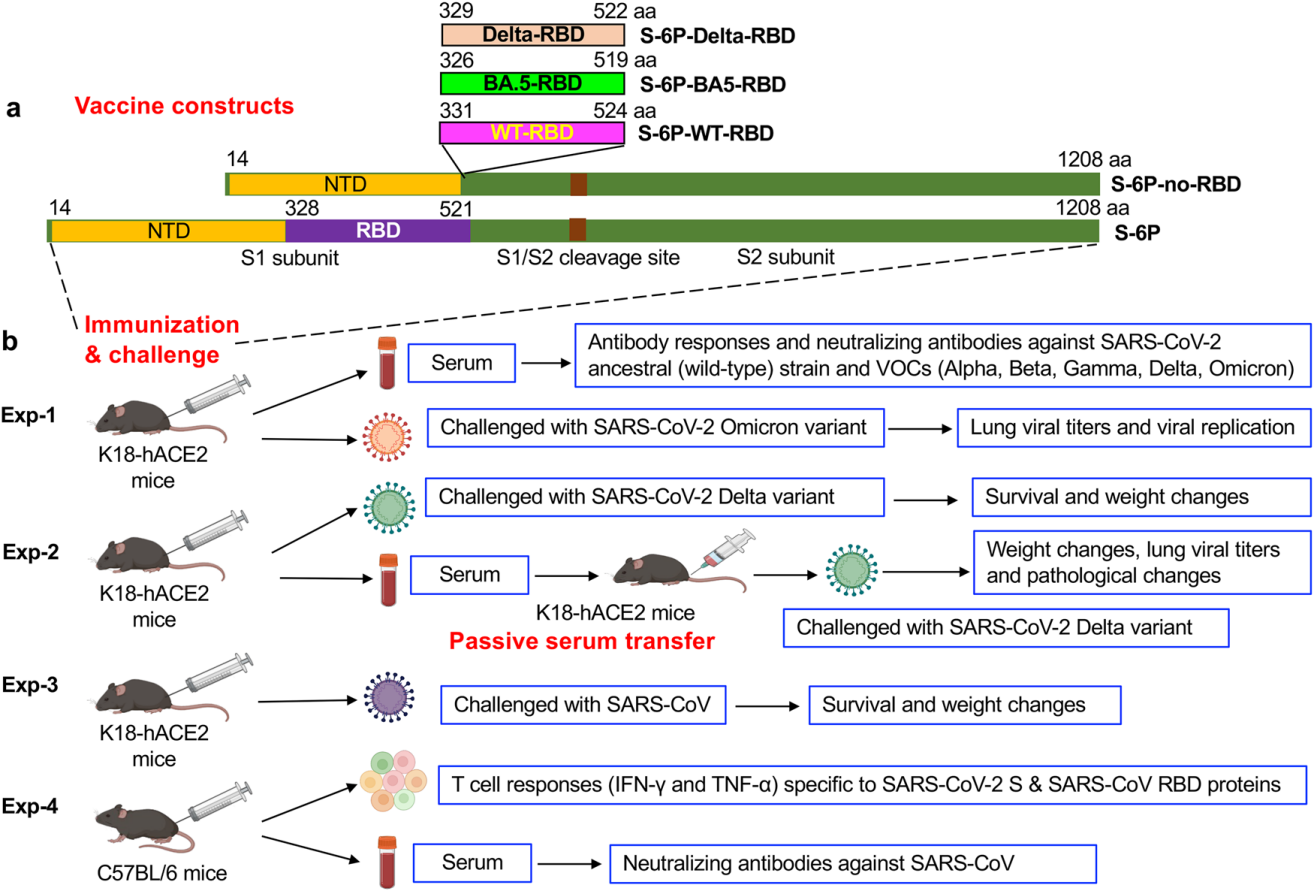
Vaccine constructs and immunization or challenge schedules. **a** S-6P-Delta-RBD, S-6P-BA5-RBD, and S-6P-WT-RBD proteins were constructed based on the backbone (truncated RBD) of the spike (S) protein of SARS-CoV-2 Omicron BA.1 subvariant (S-6P-no-RBD). The original S-6P protein (without RBD truncation) was used as a vaccine control. The amino acids (aa) were shown in each construct (the percentage of backbone and RBD: ~5.2:1). **b** Immunization and challenge schedules. K18-hACE2 (C57BL/6 background) mice were respectively immunized with the above five proteins or PBS control, plus adjuvants, and sera were collected for detection of antibody responses and neutralizing antibodies against SARS-CoV-2 wild-type and multiple strains of variants of concern (VOCs). The immunized mice were challenged with the SARS-CoV-2 Omicron variant, Delta variant, and SARS-CoV, respectively, for evaluation of protective efficacy against viral infection. The immune sera were also evaluated for passive protection against SARS-CoV-2 Delta variant and potential pathological effects. The immunized C57BL/6 mice were tested for vaccine-induced T cell responses and neutralizing antibodies. The images of mice, serum, viruses, and cells were selected from BioRender.com.

**Fig. 2:**
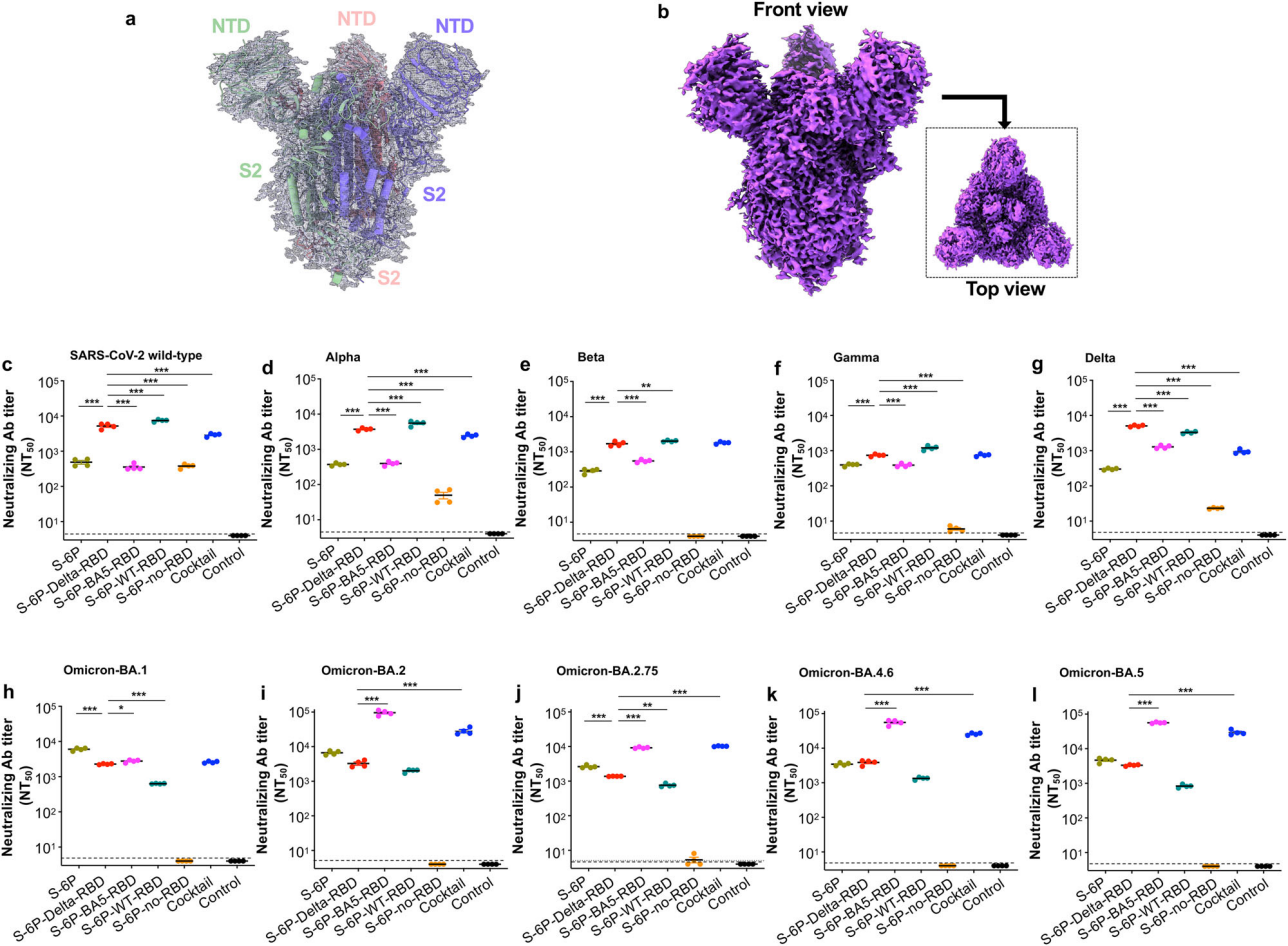
Cyro-EM structures of S-6P-no-RBD protein and neutralizing ability of subunit vaccines. **a** Overview of the cryo-EM map of the truncated spike (S) (i.e., S-6P-no-RBD) protein with structural model inside. The structure is presented in cartoons with tube helices. **b** Front and top views of the cryo-EM map. **c**–**i** Neutralizing antibodies induced by subunit vaccines against multiple variants and ancestral strain of SARS-CoV-2. The purified proteins, including S-6P, S-6P-Delta-RBD, S-6P-BA5-RBD, S-6P-WT-RBD, and S-6P-no-RBD (10 μg/mouse), or PBS control, were intramuscularly (i.m.) injected into K18-hACE2 mice in the presence of adjuvants. The cocktail consisted of the S-6P-Delta-RBD and S-6P-BA5-RBD proteins (5 μg/protein; 10 μg/mouse) with the adjuvants. The mice were boosted twice at 3-week intervals with the same immunogen and adjuvants, as described in Fig. 1. Sera collected from 10 days after the 3^rd^ immunization were evaluated for neutralizing antibodies (Abs) against pseudoviruses encoding the S protein of the ancestral (wild-type, WT) SARS-CoV-2 strain (**c**), Alpha (**d**), Beta (**e**), Gamma (**f**), and Delta (**g**) variants, as well as the Omicron BA.1 (**h**), BA.2 (**i**), BA.2.75 (**j**), BA.4.6 (**k**), and BA.5 (**l**) subvariants. The NT_50_ is expressed as 50% neutralizing Ab titers against each pseudovirus infection in 293T cells expressing human angiotensin converting enzyme-2 (hACE2/293T). The data are presented as the mean ± standard deviation of the mean (s.e.m.) of four wells from pooled sera of five mice in each group. The limit of detection for the neutralization assay was 1:5 (**c**–**l**). Ordinary one-way ANOVA (Dunnett’s multiple comparison test) was used to compare the statistical differences of neutralizing antibody titers induced by S-6P-Delta-RBD and other vaccination groups. *P < 0.05, **P < 0.01, and ***P < 0.001 designate significant differences between S-6P-Delta-RBD and other vaccination groups. The experiments were repeated twice (**c**–**e**, **h**) or three times (**f**, **g**, **i**–**l**) to confirm the results.

Four recombinant S plasmids containing the conserved backbone of S protein of SARS-CoV-2 Omicron BA.1 (a then dominant subvariant) were constructed. These include the backbone only by truncating its RBD (S-6P-no-RBD), or respectively fusing the RBD of 1) a Delta variant (S-6P-Delta-RBD), 2) an ancestral SARS-CoV-2 wild-type (S-6P-WT-RBD), or 3) an Omicron BA.5 variant (S-6P-BA5-RBD) into the above S backbone construct (Fig. 1a). The ancestral S-6P protein (without RBD truncation) was included as a control (Fig. 1a). The recombinant protein vaccines purified from transfected HEK293F cell culture supernatants had high purity and maintained strong thermal and pH stability (Supplementary Figs. 1 and 2), and they were used for analysis or subsequent immunization of mice. Cryo-EM analysis of the S-6P-no-RBD protein indicated that the S particles (with truncated RBD) formed an open conformation, and the side and top views showed that they were missing the densities corresponding to the RBD regions (Fig. 2a, b and Supplementary Fig. 3). These data demonstrate that S protein without the RBD was capable of forming conformational structures for insertion of a heterologous RBD.

### Vaccine-induced neutralizing antibodies and T-cell responses

To evaluate the ability of subunit vaccines to induce humoral immune responses, K18-human ACE2-transgenic (K18-hACE2, C57BL/6 background) mice were immunized with the respective proteins, a cocktail (combined S-6P-Delta-RBD and S-6P-BA5-RBD proteins), or PBS control, plus Aluminum Hydroxide Gel (Alum for short) and Monophosphoryl Lipid A (MPL) adjuvants, and boosted twice at 3-week intervals. Sera collected 10 days after the 3^rd^ immunization were tested for SARS-CoV-2 S/RBD-specific IgG antibodies (Fig. 1b). All vaccines induced IgG antibodies against the test proteins with varying levels, and S-6P-no-RBD induced the lowest titer of such antibodies against WT-RBD and Delta-RBD proteins (Supplementary Fig. 4a–d).

The neutralizing antibodies of related mouse sera against pseudoviruses expressing S protein of multiple SARS-CoV-2 strains were examined (Fig. 1b). S-6P-Delta-RBD elicited broad and potent neutralizing antibodies against SARS-CoV-2 WT and all VOCs tested, including Alpha, Beta, Gamma, Delta, and five Omicron subvariants (Fig. 2c–l). Notably, S-6P and S-6P-BA5-RBD induced relatively low-titer neutralizing antibodies against SARS-CoV-2 WT, and Alpha, Beta, Gamma, and Delta VOCs (Fig. 2c–g); however, S-6P-BA5-RBD elicited relatively high-titer neutralizing antibodies against the Omicron subvariants tested, particularly BA.2, BA.2.75, BA.4.6, and BA.5 (Fig. 2h–l). The neutralizing antibody titers induced by S-6P-Delta-RBD against SARS-CoV-2 WT, and Alpha, Beta, Gamma, and Delta VOCs were significantly higher (*P* < 0.001) than those induced by S-6P and S-6P-BA5-RBD (Fig. 2c–g), and were significantly higher (*P* < 0.001), or higher, than those induced by S-6P-WT-RBD against Omicron BA.1, BA.2, BA.2.75, BA.4.6, and BA.5 subvariants (Fig. 2h–l). Unlike other subunit proteins, S-6P-no-RBD induced low-titer neutralizing antibodies against SARS-CoV-2 WT (50% neutralizing antibody titer (NT_50_) < 1:500), Alpha, Delta (NT_50_ < 1:100), Gamma, or Omicron-BA.2.75 variants, but not against the other SARS-CoV-2 pseudoviruses tested (Fig. 2c–l). Of note, combined S-6P-Delta-RBD and S-6P-BA5-RBD (i.e., the cocktail) induced high levels of potent neutralizing antibodies against all SARS-CoV-2 strains tested (Fig. 2c–l). However, mice injected with adjuvant/PBS control produced no (or undetectable) neutralizing antibodies against these pseudoviruses (Fig. 2c–l).

To evaluate the ability of subunit vaccines to induce cellular immune responses, immunized C57BL/6 mice were analyzed for SARS-CoV-2 S-specific CD4^+^ and CD8^+^ T cells by flow cytometry in splenocytes 4 months after the 3^rd^ immunization (Fig. 1b). CD4^+^ and CD8^+^ T cells from all vaccination groups produced IFN-γ or TNF-α cytokines after stimulation with S-6P-no-RBD protein; cytokine levels were overall higher than those produced by splenocytes from the PBS control group (Supplementary Fig. 5a–d). Specifically, S-6P induced relatively high numbers of IFN-γ and TNF-α-secreting CD4^+^ and CD8^+^ T cells; in particular, it induced significantly higher numbers of TNF-α-secreting CD8^+^ T cells (*P* < 0.01 or *P* < 0.001) than the other immunization groups; notably, the protein cocktail further increased secretion of IFN-γ by CD8^+^ T cells (Supplementary Fig. 5a–d).

The above data demonstrate that subunit vaccines induce favorable immune responses in immunized mice. Among the single proteins tested, S-6P, particularly S-6P-BA5-RBD, alone only induced high-titer neutralizing antibodies against Omicron subvariants. By contrast, S-6P-WT-RBD, particularly S-6P-Delta-RBD, alone elicited a balanced, potent, and broad neutralizing antibody response against all SARS-CoV-2 pseudoviruses tested, with neutralizing antibody titers comparable to those induced by S-6P-Delta-RBD plus S-6P-BA5-RBD (the cocktail). Therefore, these subunit vaccines, including S-6P-Delta-RBD, have potential for development as universal COVID-19 vaccines against current and future SARS-CoV-2 VOCs.

### Protective efficacy of the vaccines against SARS-CoV-2 Delta variant

**Fig. 3:**
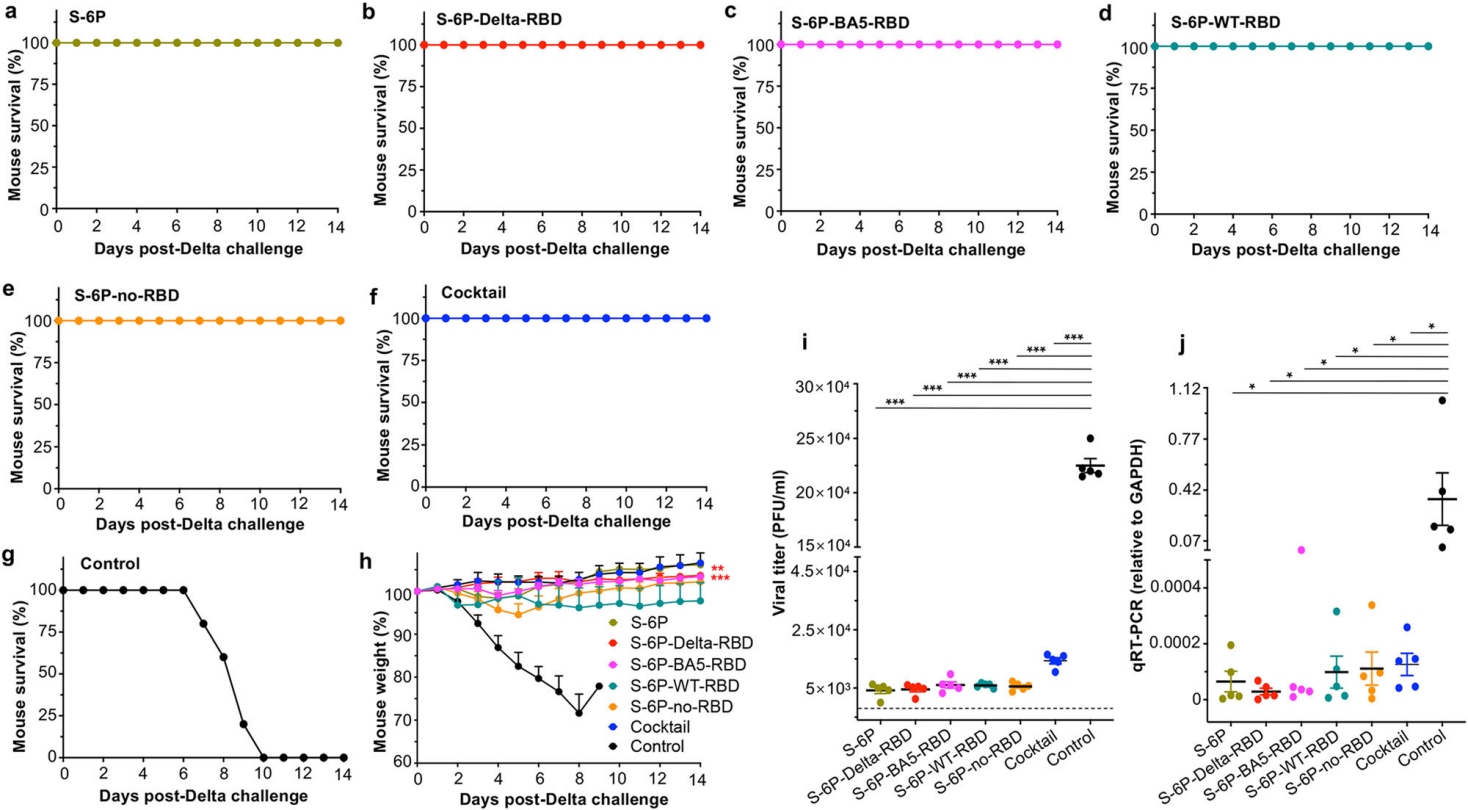
The designed subunit vaccines protected immunized K18-hACE2 mice against infection of SARS-CoV-2 Delta and Omicron variants. K18-hACE2 mice were immunized with each protein, including S-6P, S-6P-Delta-RBD, S-6P-BA5-RBD, S-6P-WT-RBD, and S-6P-no-RBD, the cocktail (combination of S-6P-Delta-RBD and S-6P-BA5-RBD proteins) in the presence of adjuvants, or PBS plus adjuvants (control), as described in Fig. 1. The immunized mice were respectively challenged with two SARS-CoV-2 variants, Delta and Omicron (BA.1 subvariant), 2 weeks after the 3^rd^ immunization. The mice challenged with a high lethal dose of Delta variant were observed for survivals (**a**–**g**) and body weight changes (**h**) for 14 days after challenge. The data (**h**) are presented as the mean + s.e.m. of five mice in each group. Ordinary one-way ANOVA (Dunnett’s multiple comparison test) was used to compare the statistical differences of weight changes between S-6P-Delta-RBD and other groups, and there are significant differences between S-6P-Delta-RBD and S-6P-WT-RBD (**P < 0.01) or PBS control group (***P < 0.001). The mice challenged with an optimal dose of Omicron variant (BA.1) were collected for lung tissues two days after viral infection, and detected for viral titers by plaque assay (**i**) and viral replication by qPCR assay (**j**). The data (**i**, **j**) are presented as the mean ± s.e.m. of five mice in each group. The limit of detection for the plaque assay was 50 plaque forming units (PFU) (**i**) and for qPCR assay was Cq value of 35 cycles (**j**). Ordinary one-way ANOVA (Tukey’s multiple comparison test) was used to compare the statistical differences of viral titers and qPCR results among different groups (**i**, **j**). *P < 0.05 and ***P < 0.001 designate significant differences among these groups. The experiments were repeated twice, with similar results.

To access the protective efficiency of subunit vaccines against lethal challenge with SARS-CoV-2 Delta VOC, immunized K18-hACE2 mice were challenged with a high lethal dose (10^4^ PFU/mouse) of this variant strain 2 weeks after the 3^rd^ vaccination, and weight loss and overall survival were monitored for 14 days (Fig. 1b). All mice receiving a single protein, or a combination, survived during the monitoring period (Fig. 3a–f). By comparison, control mice injected with adjuvant/PBS lost weight continuously, and all died by Day 10 post-challenge of Delta variant (Fig. 3g, h). In particular, no weight loss was observed in mice immunized with S-6P-Delta-RBD alone, whereas obvious or significant weight loss was observed in mice immunized with other single proteins, including S-6P, S-6P-no-RBD, S-6P-WT-RBD, and S-6P-BA5-RBD, or in mice immunized with the cocktail of proteins (Fig. 3h). These data suggest that S-6P-Delta-RBD protein alone is sufficient to prevent Delta variant-induced death and weight loss.

### Protective efficacy of the vaccines against SARS-CoV-2 Omicron variant

We examined the protective efficacy of subunit vaccines against SARS-CoV-2 Omicron VOC. BA.1, a dominant subvariant during the time of the experiment, was selected for the challenge study. Immunized K18-hACE2 mice were challenged with an optimal infectious dose of Omicron BA.1 2 weeks after the 3^rd^ immunization. Since the Omicron variant was not lethal to the test mouse model^20,21^, the viral titers and viral replication (i.e., N-gene expression) were measured 2 days post-challenge (Fig. 1b). Viral titers were highest in the lungs of control mice receiving adjuvant/PBS, which were significantly higher than those in any of the immunized groups (Fig. 3i). In particular, mice immunized with S-6P-Delta-RBD, and those with other single proteins, including S-6P, S-6P-WT-RBD, and S-6P-no-RBD, had similarly low-level viral titers in the lungs (Fig. 3i). Notably, viral titers in mice receiving combined S-6P-Delta-RBD and S-6P-BA5-RBD (the cocktail) were much higher than those in other vaccination groups (Fig. 3i). In addition, the lungs of mice receiving adjuvant/PBS showed significantly higher expression of viral N-protein than the vaccinated groups (Fig. 3j). By contrast, mice immunized with S-6P-Delta-RBD showed the lowest expression of the N-protein, followed by the mice immunized with S-6P (Fig. 3j). These data indicate that S-6P-Delta-RBD alone prevents infection and replication of SARS-CoV-2 Omicron variant in challenged mice. Therefore, this vaccine can be developed as a universal COVID-19 vaccine against SARS-CoV-2 Omicron and other variants.

### Passive protective efficacy of the vaccines against SARS-CoV-2 Delta variant

**Fig. 4:**
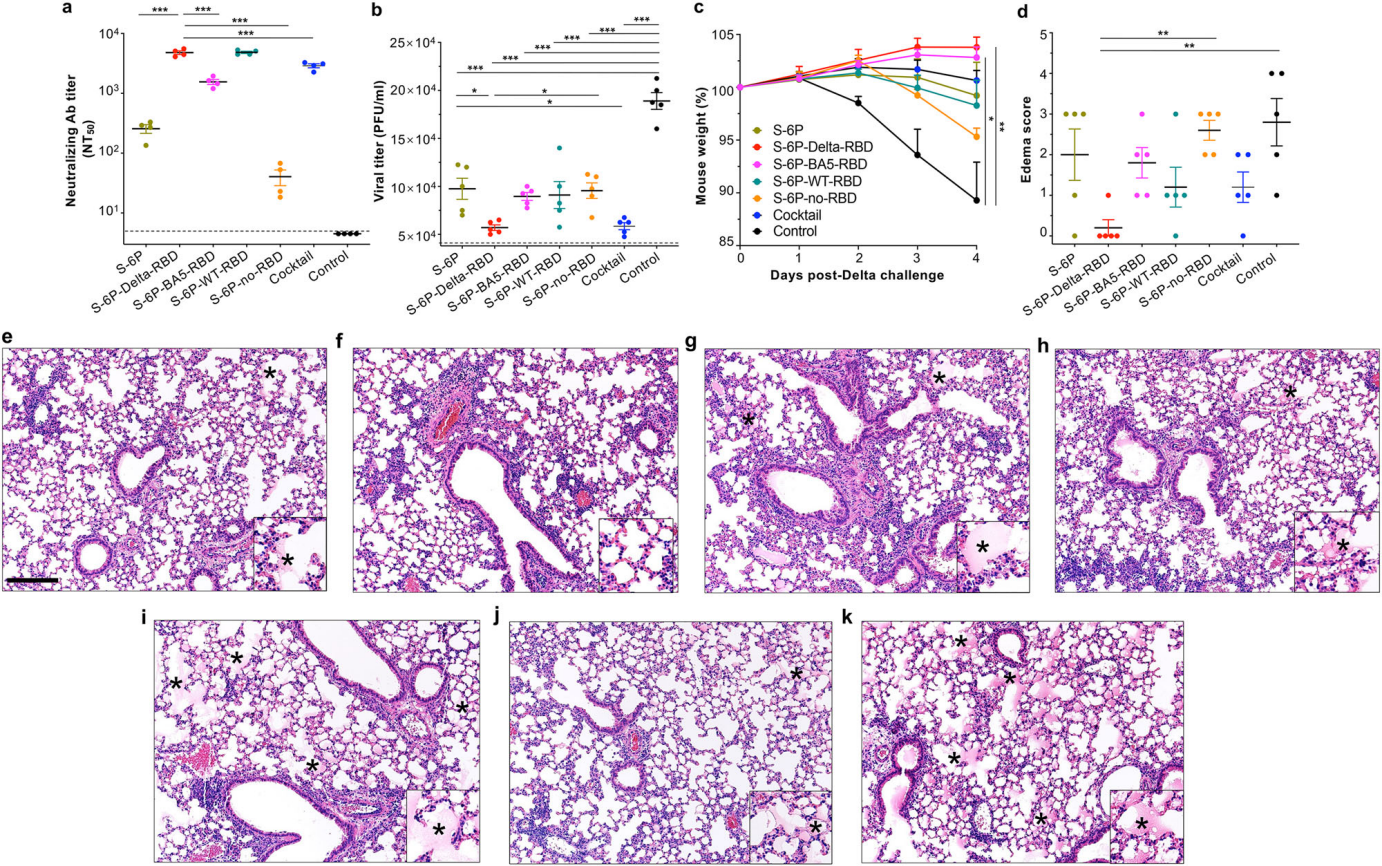
Naive K18-hACE2 mice receiving S-6P-Delta-RBD immune sera were prevented against SARS-CoV-2 infection without showing pathological effects. Naive K18-hACE2 mice were i.p. administered with pooled mouse sera (200 μl/mouse), and then i.n. challenged with SARS-CoV-2 Delta variant 6 h later, followed by evaluation of viral titers, body weight changes, and pathological changes in the lungs 4 days post-challenge. **a** 50% neutralizing antibody (Ab) titers (NT_50_) was calculated from pooled sera against SARS-CoV-2 Delta pseudovirus infection in hACE2/293T cells. **b** Viral titers of challenged mice (PFU/ml of lung tissues) was performed by plaque assay. **c** Weights were monitored for 4 days post-challenge. **d** Scoring of edema was calculated based on the H&E-stained lung tissue sections of challenged mice. Edema was scored 0 (none), 1 (< 25%), 2 (26–50%), 3 (51–75%), and 4 (> 75%) of tissue fields, respectively. Representative images are shown as H&E-stained lung tissue sections from challenged mice receiving sera of S-6P (**e**), S-6P-Delta-RBD (**f**), S-6P-BA5-RBD (**g**), S-6P-WT-RBD (**h**), S-6P-no-RBD (**i**), the cocktail (combination of S-6P-Delta-RBD and S-6P-BA5-RBD proteins) (**j**), and PBS plus adjuvants (control) (**k**). Scale bar (**e**) represents 170 μm; asterisk (*) (**e**, **g**–**k**) represents edema score. The data (**a**) are presented as mean ± s.e.m. of four wells of pooled sera. The data (**b**–**d**) are presented as mean plus s.e.m. of viral titers, weight changes, and pathological changes (edema score) from five mice in each group. The limit of detection for the neutralization assay was 1:5 (**a**) and for the plaque assay was 50 PFU (**b**). Ordinary one-way ANOVA (Dunnett’s multiple comparison test) was used to compare the statistical differences between S-6P-Delta-RBD immune sera and other vaccinated sera (**a**), and Ordinary one-way ANOVA (Tukey’s multiple comparison test) was used to compare the statistical differences among different groups (**b**–**d**). *P < 0.05, **P < 0.01), and ***P < 0.001 designate significant differences among various groups. The experiments were repeated once (**b**–**k**) or twice (**a**), with similar results.

To evaluate whether serum neutralizing antibodies generated by immunized mice play a critical role in preventing viral infection and virus-induced pathological effects, we injected naive K18-hACE2 mice with sera from each group of vaccinated mice, challenge them with SARS-CoV-2 Delta variant, and then monitored weight loss, viral titers, and pathological changes in the lungs (Fig. 1b). Neutralizing antibody titers in pooled sera were measured prior to injection into naive mice. Sera from S-6P-Delta-RBD-immunized mice showed the highest neutralizing antibody titers, followed by those from the S-6P-WT-RBD- and cocktail-immunized sera (Fig. 4a). Viral titers in mice receiving S-6P-Delta-RBD immune sera were significantly lower than those in the other groups, and no body weight loss was observed in these mice post-challenge (Fig. 4b, c). Of note, serum neutralizing antibodies generated by S-6P-Delta-RBD-immunized mice effectively protected naive recipient mice from virus-induced edema, without any obvious pathological changes (Fig. 4d, f). By contrast, S-6P-no-RBD-immunized mouse sera generated low or background neutralizing antibody titers (Fig. 4a). Thus, naive mice receiving these sera had significantly higher viral titers in the lungs, lost weight consistently after challenge, and suffered more edema than mice receiving other immune sera (Fig. 4b–d, i). Overall, the higher the neutralizing antibody level in transferred sera, the lower viral titer and the fewer pathological changes were observed in the lungs of recipient naive mice (Fig. 4a–k). These data demonstrate that serum neutralizing antibodies generated by S-6P-Delta-RBD-immunized mice are critical for protecting mice from subsequent challenge with the Delta variant, and that the protective efficacy correlated positively with the neutralizing antibody titers.

### Protective efficacy of the vaccines against SARS-CoV

**Fig. 5:**
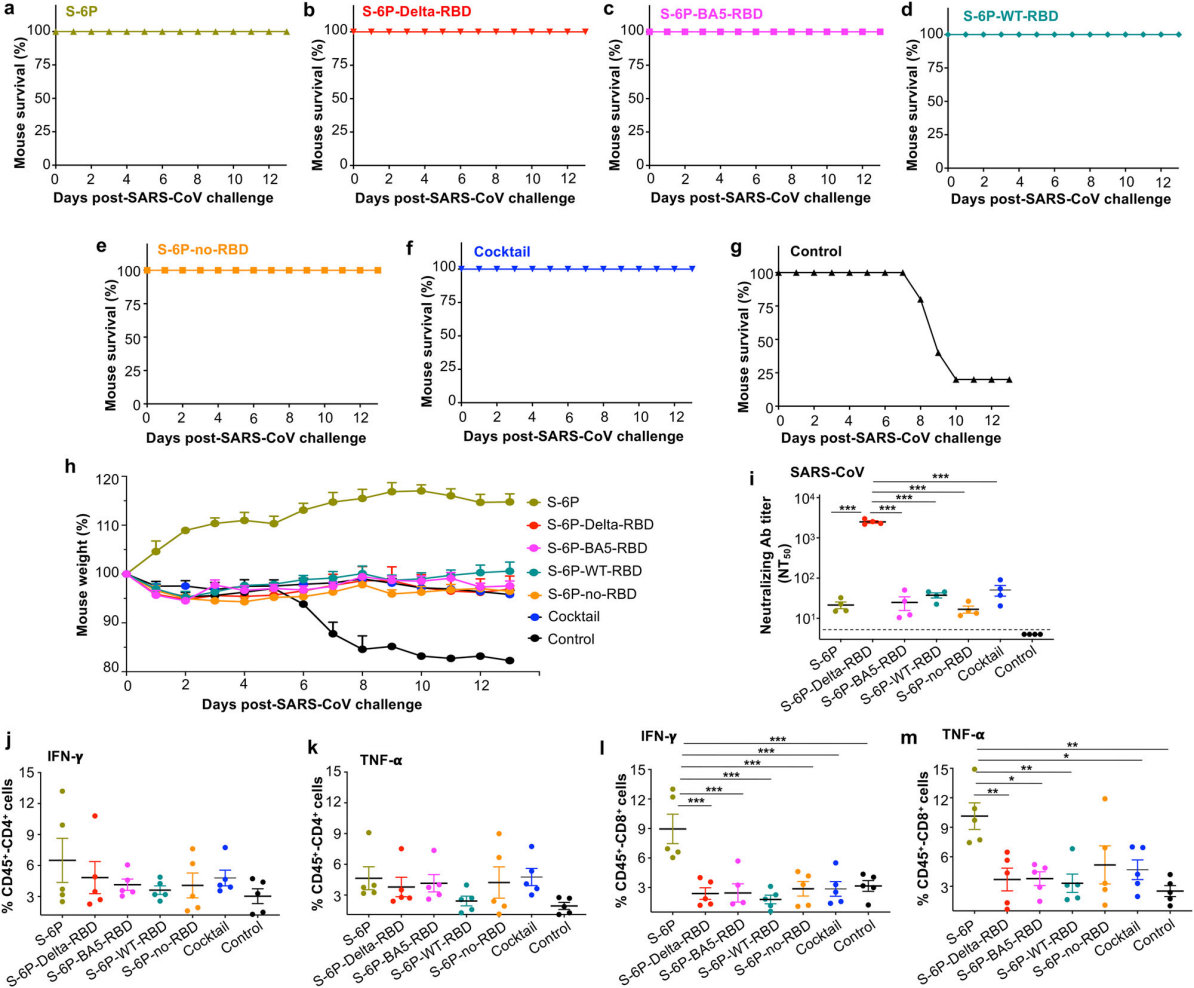
The designed subunit vaccines induced SARS-CoV-specific immune responses and protected K18-hACE2 mice against SARS-CoV infection. K18-hACE2 mice were immunized with S-6P, S-6P-Delta-RBD, S-6P-BA5-RBD, S-6P-WT-RBD, or S-6P-no-RBD, the cocktail (combination of S-6P-Delta-RBD and S-6P-BA5-RBD proteins) in the presence of adjuvants, or PBS plus adjuvants (control), as described in Fig. 1. The immunized mice were challenged with a lethal dose of SARS-CoV (MA15 strain) 2 weeks after the 3^rd^ immunization. The challenged mice were observed for overall survival (**a**–**g**) and weight changes (**h**) for 13 days after SARS-CoV challenge. In a separate experiment, C57BL/6 mice were immunized as described above, collected for sera and splenocytes 4 months after the 3^rd^ immunization, and detected for neutralizing antibodies against pseudotyped SARS-CoV in hACE2/293T cells (**i**), as well as IFN-γ or TNF-α-producing CD4^+^ (**j**, **k**) and CD8^+^ (**l**, **m**) T cells, respectively. NT_50_ is expressed as 50% neutralizing antibody (Ab) titers of sera against SARS-CoV pseudovirus in hACE2/293T cells, and the data (**i**) are presented as the mean plus s.e.m. of four wells from pooled sera of five mice in each group. The limit of detection for the neutralization assay was 1:5. The splenocytes were stimulated with a Fc-fused SARS-CoV RBD protein, and the secreted cytokines were measured by flow cytometry. The data (**j**–**m**) are presented as the mean plus s.e.m. of five mice in each group. Ordinary one-way ANOVA (Dunnett’s multiple comparison test) was used to compare the statistical differences between S-6P-Delta-RBD and other vaccinated groups (**i**), and Ordinary one-way ANOVA (Tukey’s multiple comparison test) was used to compare the statistical differences among different groups (**l**, **m**). *P < 0.05, **P < 0.01, and ***P < 0.001 designate significant differences among various groups. The experiments were repeated twice, with similar results.

To evaluate the protective efficacy of subunit vaccines against SARS-CoV, immunized K18-hACE2 mice were challenged with a lethal dose (200 PFU/mouse) of SARS-CoV MA15 strain 2 weeks after the 3^rd^ immunization, and overall survival and body weight changes were monitored for 13 days post-challenge (Fig. 1b). All mice immunized with a single protein, including S-6P, S-6P-Delta-RBD, S-6P-BA5-RBD, S-6P-WT-RBD, or S-6P-no-RBD (Fig. 5a–e), and all mice receiving the two combined proteins (the cocktail) (Fig. 5f), survived without weight loss (Fig. 5h). By contrast, the control mice receiving adjuvant/PBS had consistent weight loss and 80% of them died by Day 10 post-challenge (Fig. 5g, h).

To elucidate the protective mechanisms of these subunit vaccines, splenocytes and serum samples were harvested from immunized C57BL/6 mice 4 months after the 3^rd^ immunization, and analyzed for SARS-CoV RBD-specific CD4^+^ and CD8^+^ T cells and neutralizing antibodies against pseudotyped SARS-CoV (Fig. 1b). S-6P-Delta-RBD protein alone elicited the highest level of such neutralizing antibodies, which were significantly higher (*P* < 0.001) than those induced by the other vaccination groups, including S-6P, S-6P-BA5-RBD, S-6P-WT-RBD, or S-6P-no-RBD alone, and the cocktail proteins (Fig. 5i). Whereas S-6P-Delta-RBD, S-6P-BA5-RBD, and the cocktail induced similar levels of IFN-γ or TNF-α-producing CD4^+^ T cells (Fig. 5j, k), S-6P elicited higher or significantly higher levels of IFN-γ and TNF-α-producing CD8^+^ T cells than the other vaccination groups (Fig. 5l, m), potentially explaining why mice maintained increased body weight post-SARS-CoV challenge (Fig. 5h). These data indicate that single proteins, or the combined S-6P-Delta-RBD and S-6P-BA5-RBD proteins, completely protected vaccinated mice against SARS-CoV-induced death and weight loss, and that both neutralizing antibodies and T cell responses likely play a role in protection against SARS-CoV infection. Thus, these subunit vaccines, particularly S-6P-Delta-RBD, have potential for development as universal vaccines against SARS-CoV-2 and SARS-CoV.

## Discussion

A variety of COVID-19 vaccines have been developed, several of which (including at least two mRNA vaccines, one adjuvanted nanoparticle vaccine, and one viral vector vaccine) have been approved or authorized for emergency use in humans^22–24^. These vaccines, most of which are based on the S protein of SARS-CoV-2, have made significant contributions to alleviating SARS-CoV-2 infection and stalling the COVID-19 pandemic^25–27^. However, SARS-CoV-2 (particularly the RBD fragment) mutates much more quickly than any other known coronaviruses; this led to rapid emergence of at least five VOCs (Alpha, Beta, Gamma, Delta, and Omicron), with Omicron itself having multiple subvariants^3,28,29^. Thus, the virus poses a continuous threat to global public health. These variants, especially Omicron subvariants, show significant resistance to the currently developed or approved COVID-19 vaccines that target the ancestral or early-emerging strains^30,31^. Other coronaviruses, such as SARS-CoV and SARS-related coronaviruses, share the same ACE2 receptor as SARS-CoV-2^32,33^, meaning that they also have pandemic potential. Currently, no approved vaccines target these coronaviruses. Thus, pan-SARS-CoV-2 or universal coronavirus vaccines against multiple SARS-CoV-2 variants, as well as other coronaviruses such as SARS-CoV or SARS-related coronaviruses, are needed.

The S protein of SARS-CoV-2 is a critical target for development of potent vaccines that elicit antibodies with strong neutralizing activity against viruses. The majority of the currently identified neutralizing epitopes are located in the RBD region of the S protein^34^. While most vaccines targeting the ancestral or variant S protein, including the respective RBDs, are effective against homologous strains, they have reduced, or completely absent, neutralizing activity against heterologous variants^5,35^. The RBD of the Omicron variant S protein has more than 20 amino acid mutations compared with the ancestral or the earlier RBDs^3,19^. Compared to the RBD, the other regions of the S protein are relatively conserved among all SARS-CoV-2 strains. We hypothesized that universal COVID-19 vaccines can be developed based on the conserved backbone (truncated RBD) of SARS-CoV-2 S protein, and replacement of the highly mutated Omicron RBD with a relatively conserved RBD of an earlier variant or the ancestral RBD of SARS-CoV-2.

Here, we constructed several subunit vaccines by respectively inserting heterologous SARS-CoV-2 RBDs with no or less mutations to the conserved backbone of the Omicron BA.1 S protein. The RBD-truncated S backbone protein forms conformational structures for insertion of the heterologous RBD fragment. Among the proteins generated, the S-6P-Delta-RBD protein induced a broad and potent neutralizing antibody response against all SARS-CoV-2 VOCs tested, as well as SARS-CoV-2 ancestral strain. The RBD of the SARS-CoV-2 Delta variant has been shown to induce neutralizing antibodies against SARS-CoV-2 VOCs, which are higher than those induced by the ancestral RBD^36^. Our previous study also demonstrated that Delta-RBD elicited broadly neutralizing antibodies against several SARS-CoV-2 VOCs, including Delta and Omicron, providing better protection of transgenic mice than Omicron-RBD-containing S protein from lethal virus challenge^37^. Indeed, we found that insertion of the Delta-RBD to the conserved Omicron BA.1 S backbone increased production of high-titer neutralizing antibodies against multiple SARS-CoV-2 VOCs (including Omicron subvariants), and SARS-CoV, which were significantly more strongly than those induced by the S-6P-WT-RBD (containing the BA.1 S backbone and ancestral RBD) or S-6P-BA5-RBD (containing the BA.1 S backbone and BA.5 RBD) protein. The increased cross-neutralizing antibodies induced by S-6P-Delta-RBD may be due to the mutant residue identified on the Delta-RBD, or exposure of additional neutralizing epitopes, which will be warranted for future studies.

S-6P-Delta-RBD completely protected animals against subsequent challenge with SARS-CoV-2 Delta and Omicron BA.1 VOCs, as well as SARS-CoV, resulting in similar or better protection than the other proteins tested. Interestingly, the S-6P-Delta-RBD and S-6P-BA5-RBD combination was less effective in protecting mice against the SARS-CoV-2 Omicron variant replication and infection than the S-6P-Delta-RBD and S-6P-BA5-RBD proteins alone. This might be potentially due to the reduced concentration of each single protein in the inoculum, or because the neutralizing epitopes within one or two protein components may not be completely exposed in the combined vaccines, which potentially affect their overall protective efficacy. Thus, combinations of two or more vaccines might not be ideal for maximal inhibition of Omicron replication. Instead, combining the BA.1 S backbone with the Delta RBD is an ideal vaccine against multiple SARS-CoV-2 strains and SARS-CoV. The present study used a previously optimized concentration (10 μg) of the proteins for immunization^18,38^. Future studies will evaluate the neutralizing activity and protective efficacy of these subunit vaccines at lower doses.

Neutralizing antibodies play a critical role in protection against infection by SARS-CoV-2 and SARS-CoV^9,39,40^. The potential protective mechanism of S-6P-Delta-RBD against the Delta variant could be mainly due to the vaccine-induced neutralizing antibodies, as high-titer serum neutralizing antibodies from S-6P-Delta-RBD-immunized mice led to complete protection of naive mice from virus challenge without pathological effects. In comparison, lower-titer or lack of neutralizing antibodies induced by S-6P-no-RBD resulted in reduced protection and pathological changes in the lungs. We are aware that proteins that do not induce neutralizing antibodies, or only induce low-titer neutralizing antibodies, triggered cellular immune responses specific to the backbone of SARS-CoV-2 S or the RBD of SARS-CoV that may provide some protection against SARS-CoV-2 and SARS-CoV infections. Therefore, although S-6P-no-RBD did not induce, or elicited low-titer, neutralizing antibodies against most of the pseudoviruses tested, it did elicit specific T-cell responses, leading to the protective efficacy against viral infection. Among all proteins tested, S-6P induced the highest level of SARS-CoV RBD-specific T-cell responses, preventing SARS-CoV-caused weight loss. This could be potentially due to the fact that the RBD of S-6P contains more SARS-CoV RBD-specific T-cell epitopes compared with the other inserted RBDs. Overall, neutralizing antibody and/or T cell responses are needed for effective protection against the SARS-CoV-2 and SARS-CoV strains tested.

The rationale for selection of the S protein of an early Omicron subvariant, BA.1, as the backbone of the vaccine constructs is that this subvariant was dominant during the time of experiments; also, the sequence of this S backbone region is highly conserved among the S proteins of different SARS-CoV-2 strains, including Omicron subvariants. The conserved S backbone regions of other SARS-CoV-2 variants are also warranted as future vaccine constructs. Notably, the protective efficacy of the designed vaccines against SARS-CoV Omicron infection was only performed using Omicron BA.1 subvariant. Due to their ability to induce broadly neutralizing activity and cross-protective T-cell responses against multiple SARS-CoV-2 variants and SARS-CoV tested in this study, these vaccines are expected to protect against other Omicron subvariants, which will be tested in future studies.

To summarize, we designed several subunit vaccines based on the conserved backbone of the S protein, which respectively encoding different versions of the RBD of SARS-CoV-2 variants or the wild-type strain. Among those tested, the vaccine containing the Delta variant RBD protein induced highly potent and broadly neutralizing antibodies that provided complete protection against all SARS-CoV-2 strains tested, as well as SARS-CoV. This protein has great potential for development as a universal vaccine against SARS-CoV-2 and SARS-related coronaviruses to contain the COVID-19 pandemic and relieve the threat from SARS-CoV or SARS-related coronaviruses. Such a tool is needed to reduce the threat from current and future SARS-CoV-2 and SARS-related coronaviruses with pandemic potential. Future studies will be conducted to evaluate the protective efficacy of this vaccine in other animal models (such as Golden Syrian hamsters and then non-human primates) or SARS-related beta-coronavirus models, and evaluate the vaccine-induced long-term durability of the immune responses and protective efficacy against viral infection.

## Methods

### Experimental design and vaccine preparation

The subunit vaccines were prepared as described below^18^. Specifically, the DNA sequence of SARS-CoV-2 Omicron BA.1 variant containing a C-terminal Foldon trimerization domain and a His_6_ tag (S-6P) was amplified by PCR based on a codon-optimized plasmid expressing the S protein of Omicron BA.1 variant of SARS-CoV-2 (GISAID accession number EPI_ISL_6795835) with HexaPro sequences (a mutated furin cleavage site and 6 proline substitutions). The amplified PCR fragment was inserted into a pLenti mammalian cell expression vector. The S-6P-no-RBD was constructed by truncating the RBD fragment of the above S-6P sequence, and ligating using ClonExpress MultiS One Step Cloning kit (Cellagen Technology). The S-6P-Delta-RBD, S-6P-BA5-RBD, and S-6P-WT-RBD were constructed by inserting the respective RBD fragment of SARS-CoV-2 Delta variant (GISAID accession number EPI_ISL_7178410), Omicron BA.5 subvariant (GISAID accession number EPI_ISL_12043290), or the ancestral SARS-CoV-2 strain (GenBank accession number QHR63250.2) using the above Cloning kit. Each recombinant plasmid was confirmed for correct sequences by sequencing analysis, and transfected into HEK293F cells, followed by purification of the related proteins from the cell culture supernatants using Ni-NTA Superflow (Qiagen).

### SDS-PAGE of the purified proteins

The purified proteins were analyzed by SDS-PAGE. Specifically, the proteins were mixed with Laemmli SDS-PAGE Sample buffer (Bio-Rad), and separated by 8% SDS-PAGE Gel in the presence of Tris-Glycine running buffer, followed by staining using SimplyBlue SafeStain buffer (Thermo Fisher Scientific).

### Thermal shift assay of the purified proteins

The thermal stability of the expressed proteins at variable pH values was performed using the protein gel stain reagent and real-time PCR machine based on the manufacturer’s instruction. Specifically, 10 µl of each protein was added into a 96-well PCR plate, followed by addition of 2.5 µl of 50× SYPRO Ruby Protein Gel Stain (Sigma-Aldrich). In total, 12.5 µl of Tris buffers at specific pH values was then added to each well. The melt temperature of the proteins in each well was measured by CFX Opus 96 Real-Time PCR System instrument (Bio-Rad).

### Cryo-EM grid preparation and data acquisition

The isolated S-6P-no-RBD protein (4 µl at 3.78 μM) was applied to freshly glow-discharged Quantifoil R1.2/1.3 300-mesh copper grids (EM Sciences), and then blotted for 4 s at 4 °C under 100% chamber humidity and plunge-frozen in liquid ethane using a Vitrobot Mark IV (FEI). Cryo-EM data were collected using Latitude-S (Gatan) on a Titan Krios electron microscope (Thermo Fisher Scientific) equipped with a K3 direct electron detector with a Biocontinuum energy filter (Gatan) in CDS mode at the Hormel Institute, University of Minnesota. The movies were collected at a nominal magnification of ×130,000 (corresponding to 0.664 Å per pixel), a 20 eV slit width, a dose rate of 21 e– per Å^2^ per second, and a total dose of 42 e − /Å^2^. The statistics of cryo-EM data collection are summarized in Supplementary Table 1.

### Image processing

Cryo-EM data were processed using cryoSPARC v4.0.3^41^, and the data processing procedures are outlined in Supplementary Fig. 3. Dose-fractionated movies were first subjected to Patch motion correction and Patch CTF estimation with MotionCor2^42^ and CTFFIND-4.1.13^43^, respectively. Images with the defocus values outside of −0.6 to −2.8 μm or the CTF fit resolutions worse than 5 Å were excluded from the further steps. Particles were picked using both Blob picker and Template picker accompanied with removing duplicate particles. Three rounds of 2D classifications were applied to remove junk particles and particles (319, 289) extracted from the good 2D classes were used for Ab-initio Reconstruction of four maps and then for the heterogeneous refinements. The good 3D class (171, 788 particles) was finally subjected to further homogeneous, non-uniform and CTF refinements to generate a 2.8 Å resolution final map with applying C3 symmetry. Map resolution was determined by gold-standard Fourier shell correlation (FSC) at 0.143 between the two half-maps. Local resolution variation was estimated from the two half-maps in cryoSPARC v4.0.3.

### Model building and refinement

Initial model building of the S-6P-no-RBD was performed in Coot-0.8.9^44^ using PDB 7TGW without RBD domains as the starting model. Several rounds of refinement in Phenix-1.16^45^ and manually building in Coot-0.8.9 were performed until the final reliable models were obtained. The final model has good stereochemistry by evaluation in MolProbity^46^. The statistics of 3D reconstruction and model refinement are shown in Supplementary Table 1. Figures were generated using UCSF Chimera X v0.93^47^.

### Construction of recombinant plasmids

The recombinant plasmids were constructed as described below^18^. Specifically, the recombinant plasmids encoding the S protein of SARS-CoV-2 ancestral wild-type (WT) strain (GenBank accession number QHR63250.2), SARS-CoV original strain (GenBank accession number AY274119), and SARS-CoV-2 Alpha variant (GISAID accession number EPI_ISL_718813) were constructed by inserting each DNA sequence into a pcDNA3.1/V5-His-TOPO vector. SARS-CoV-2 Omicron BA.1 (GISAID accession number EPI_ISL_6795835), BA.2 (GISAID accession number EPI_ISL_12030355), BA.2.75 (GISAID accession number EPI_ISL**_**14384334), BA.4.6 (GISAID accession number EPI_ISL_14288784), and BA.5 (GISAID accession number EPI_ISL_12043290), as well as recombinant plasmids expressing each S protein of Beta, Gamma, and Delta variants, were constructed using a multi-site-directed mutagenesis kit, and the mutations in the RBD region were included in each construct. The sequence-confirmed plasmids were used for generation of pseudoviruses as described below.

### Pseudovirus generation and neutralization assay

The pseudoviruses were generated as described below^48–50^. Specifically, each of the above recombinant plasmid was co-transfected with the pLenti-CMV-luciferase and PS-PAX2 plasmids into 293T cells using a PEI transfection method. Pseudovirus-containing culture supernatants were collected 72 h after transfection, and incubated with serially diluted mouse sera (2–4-fold serial dilutions for serum neutralizing antibodies against different strains of SARS-CoV-2 pseudovirus, and two-fold serial dilutions for serum neutralizing antibodies against SARS-CoV pseudovirus) at 37 °C for 1 h. The virus-serum mixture was then added to 293T cells expressing human ACE2 receptor (hACE2/293 T). 24 h later, fresh medium was added to the cells, which were further cultured for 48 h. The cells were then sequentially incubated with cell lysis buffer and luciferase substrate (Promega), and measured for relative luciferase activity using Cytation 7 Microplate Multi-Mode Reader and Gen5 software. 50% pseudovirus neutralization was calculated as NT_50_.

### Ethics statement

Male and female K18-hACE2 transgenic mice (6–8-week-old) and C57BL/6 mice (8–10-week-old) were used in this study, and they were randomly assigned to each group. The animal protocols were approved by the Institutional Animal Care and Use Committees (IACUC) of Georgia State University and University of Iowa. All mouse-related experiments were conducted by strictly following our approved protocols and the Guidelines for the Care and Use of Laboratory Animals of National Institutes of Health. Mice reaching 25% weight loss with significant clinical symptoms, or 30% weight loss, were humanely euthanized by cervical dislocation under anesthesia.

### Animal immunization and serum collection

The K18-hACE2 mice were i.m. immunized with each protein (10 μg/mouse) or PBS control plus Alum (500 μg/mouse) and MPL (10 μg/mouse) adjuvants (InvivoGen). The selection of the above adjuvants and the doses of the proteins was based on our previously optimized schedules^18,37,38^. The cocktail is the combination of S-6P-Delta-RBD and S-6P-BA5-RBD proteins (5 μg for each protein, 10 μg/mouse). These mice were further boosted twice with the same immunogen and adjuvants every 3 weeks. The sera were collected from each mouse 10 days after the 3^rd^ immunization and measured for neutralizing antibodies against SARS-CoV-2 and SARS-CoV (as described above), and IgG antibody responses (as described below), or pooled for passive transfer to naive mice for subsequent challenge studies (as described below). In a separate experiment, C57BL/6 mice were immunized as described above, and sera and splenocytes were collected 4 months after the 3^rd^ immunization for detection of neutralizing antibodies (as described above) and T cell responses (as described below).

### ELISA

ELISA was carried out to test S/RBD-specific IgG antibodies in immunized mouse sera^38,48^. Specifically, ELISA plates were coated with the respective SARS-CoV-2 S or RBD protein (1 μg/ml) at 4 °C overnight, and blocked at 37 °C for 2 h, with 2% fat-free milk in PBS containing 0.05% Tween-20 (PBST). After washing with PBST for five times, the plates were sequentially incubated with 3-fold serially diluted sera, and horseradish peroxidase (HRP)-conjugated anti-mouse IgG (Fab specific) antibody (1:60,000 dilution; Sigma, #A9917) at 37 °C for 1 h. The plates were washed again as described above, incubated with TMB (3,3’,5,5’-Tetramethylbenzidine) substrate (Sigma, #T4319), and stopped for reaction by H_2_SO_4_ (1 N). The absorbance at 450 nm (A450) was measured using Cytation 7 Microplate Multi-Mode Reader.

### Flow cytometry analysis

Flow cytometry was carried out to analyze SARS-CoV-2 or SARS-CoV-specific CD4^+^ and CD8^+^ T cell responses in the mouse splenocytes^51,52^. Briefly, splenocytes (1 × 10^6^ cells/ml, 200 µl/well) were incubated with 5 μg/ml of S-6P-no-RBD (for SARS-CoV-2) or a Fc-fused SARS-CoV RBD (for SARS-CoV)^50^ protein diluted in RPMI 1640 medium containing 10% FBS and mouse IL-2 (R&D Systems, 1 ng/ml) at 37 °C. After 41 h, the cells were restimulated as above for 1 h, followed by incubation with Brefeldin A (5 μg/ml; Biolegend, #420601) and BD GolgiStop™ Protein Transport Inhibitor (containing Monensin) (1:1500 dilution; BD Biosciences, #554724) for another 6 h. After washing with PBS, the cells were stained with Fixable Viability Dye eFluor™ 660 (1:1000 dilution; eBioscience, #65-0864-14) to exclude dead cells. After incubation with TruStain FcX plus (1:200 dilution; Bioligand, #156604) to block Fc receptors, the cells were then stained with anti-mouse CD45-PE-Cy7 (1:333 dilution; BD Biosciences, #552848) and anti-mouse-CD8-FITC (1:200 dilution; BD Biosciences, #553031) or anti-mouse-CD4-FITC (1:200 dilution; Biolegend, #100406) antibodies. After fixation and permeabilization (Fixation/Permeabilization Kit, BD Biosciences, #554714), the cells were stained with anti-mouse-IFN-γ-PE (1:100 dilution; Biolegend, #505808) or anti-mouse-TNF-α-BV421 (1:100 dilution; Biolegend, #506328) antibody, and analyzed by CytoFLEX flow cytometer (Beckman Coulter Life Sciences). The gating strategy and representative plot of flow cytometry are shown in Supplementary Figs. 6 and 7.

### Challenge of immunized mice with SARS-CoV-2 or SARS-CoV

Three separate challenge experiments were performed 2 weeks after the 3^rd^ immunization as described below^18,38,53^. (1) The immunized mice were intranasally (i.n.) infected with SARS-CoV-2 Delta variant (a high lethal dose of 10^4^ plaque forming units (PFU)/mouse, 50 μl/mouse), and observed for body weight changes and survival for 14 days after challenge. (2) The immunized mice were i.n. infected with SARS-CoV-2 Omicron variant (BA.1 subvariant, an optimal infectious dose of 10^5^ PFU/mouse, 50 μl/mouse). The mice were then sacrificed two days after infection, and the lungs were collected for measurement of viral titers and replication. (3) The immunized mice were i.n. infected with SARS-CoV MA15 strain (a lethal dose of 200 PFU/mouse, 50 μl/mouse), and observed for body weight changes and survival for 13 days after infection (note: the reason that these mice were not observed for 14 days post-SARS-CoV challenge, as for post-SARS-CoV-2 Delta variant challenge, was due to closure of the BSL-3 laboratory for yearly maintenance).

### Challenge of naive mice receiving immune sera with SARS-CoV-2 Delta variant

Pooled mouse sera (200 μl/mouse) from the respective immunization groups were intraperitoneally (i.p.) administered to naive mice^54^. Six hours after serum administration, the mice were i.n. infected with SARS-CoV-2 Delta variant (5 × 10^3^ PFU/mouse: a dose optimized for evaluating viral titers and pathological changes in the lungs; 50 μl/mouse). Four days after the viral infection, the mice were euthanized for lung collection. Half lungs were collected in PBS for measurement of viral titers by plaque assay, and the other half were collected in Zinc formalin for pathological analysis, as described below.

### Viral titer detection

Lungs collected from SARS-CoV-2 Omicron or Delta variant-challenged mice were measured for viral titers by a standard plaque assay as described below^53^. Specifically, lung tissues were homogenized and centrifuged, and the supernatants were diluted in DMEM cell culture medium, followed by incubation with Vero E6 cells at 37 °C for 1 h. The inoculation was removed, and the cells were then overlaid with 0.6% agarose and cultured for three days. After removing the overlays, the cells were stained with 0.1% crystal violet to show plaques. Viral titers were calculated as PFU/ml of mouse lung tissues.

### RNA isolation and qRT-PCR

Lungs collected from SARS-CoV-2 Omicron variant-challenged mice were also measured for viral replication as described below^53^. Specifically, RNA was extracted from virus-infected mouse lungs using Invitrogen TRIzol reagent (Thermo Fisher Scientific) according to the manufacturer’s instructions. In all, 1 μg of total RNA was used as template for the first strand of cDNA. The resulting cDNA was subjected to amplification of selected genes by real-time quantitative PCR (qRT-PCR) using Power SYBR Green PCR Master Mix (Applied Biosystems). Viral nucleocapsid (N) gene was detected using nCOV_N1 primer (Integrated DNA Technologies, 10007031). The expression levels were normalized to glyceraldehyde-3-phosphate dehydrogenase (GAPDH) using the following threshold cycle (CT) equation: ΔCT = CT of the gene of interest − CT of GAPDH. The results are expressed as a ratio to GAPDH calculated as 2^−ΔCT^.

### Pathological analysis of lung tissues

Lungs collected from SARS-CoV-2 Delta variant-challenged mice were analyzed for pathological effects. Specifically, paraffin-embedded lung tissue sections were stained using the hematoxylin and eosin (H&E) method, and the relevant slides were examined for pathological effects using a grouped masking approach^55^. Edema distribution in the lungs were ordinally numbered^56^, with the scores being reported as 0 (none), 1 (< 25%), 2 (26–50%), 3 (51–75%), and 4 (> 75%) of tissue fields. High resolution images were taken using a BX53 Microscope (DP73 digital camera) and CellSens Dimension software (Olympus).

### Statistical analysis

GraphPad Prism 9.0.2 statistical software was used to calculate statistical significances among various vaccination groups. Ordinary one-way ANOVA (Dunnett’s multiple comparison test) was performed to assess statistical significances between S-6P-Delta-RBD and other groups, and Ordinary one-way ANOVA (Tukey’s multiple comparison test) was performed to assess statistical significances among different groups. The respective statistical tests are shown in the related figure legends. *, **, and *** designate *P* < 0.05, *P* < 0.01, and *P* < 0.001, respectively.

### Supplementary information

Supplemental material

## Data Availability

All data and results needed to evaluate the conclusions in this study are presented in the paper and/or the supplementary materials. Materials used in this study will be made available under a Material Transfer Agreement. The atomic model generated in this study has been deposited into the Protein Data Bank (PDB: 8THF). The corresponding cryo-EM density map generated in this study has been deposited into the Electron Microscopy Data Bank (EMDB: EMD-41260). There is no special code used in this paper.
