# Supplementary material for: Universal subunit vaccine protects against multiple SARS-CoV-2 variants and SARS-CoV
Source: NPJ Vaccines. 2024 Jul 25;9:133. doi: 10.1038/s41541-024-00922-z (PMC11272943; doi:10.1038/s41541-024-00922-z)
Supplement: Supplementary file 1 — Supplemental material [file 41541_2024_922_MOESM1_ESM.pdf]

Supplementary Materials

Supplementary Figures

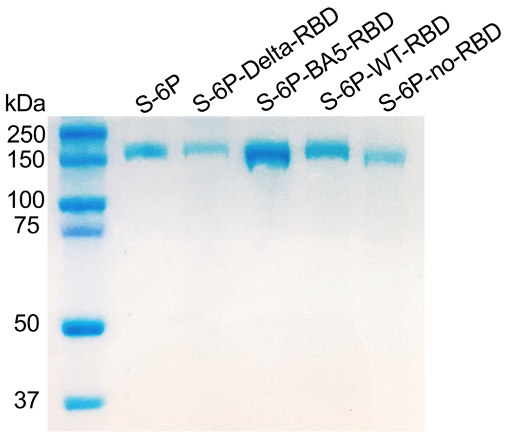

Supplementary Figure 1: SDS-PAGE of the purified proteins.

Each protein was incubated with an SDS-PAGE sample buffer and stained with SimplyBlue SafeStain buffer. The molecular weight marker (kDa) of the proteins is shown on the left. The protein samples were processed in parallel and the gel was from the same experiment.

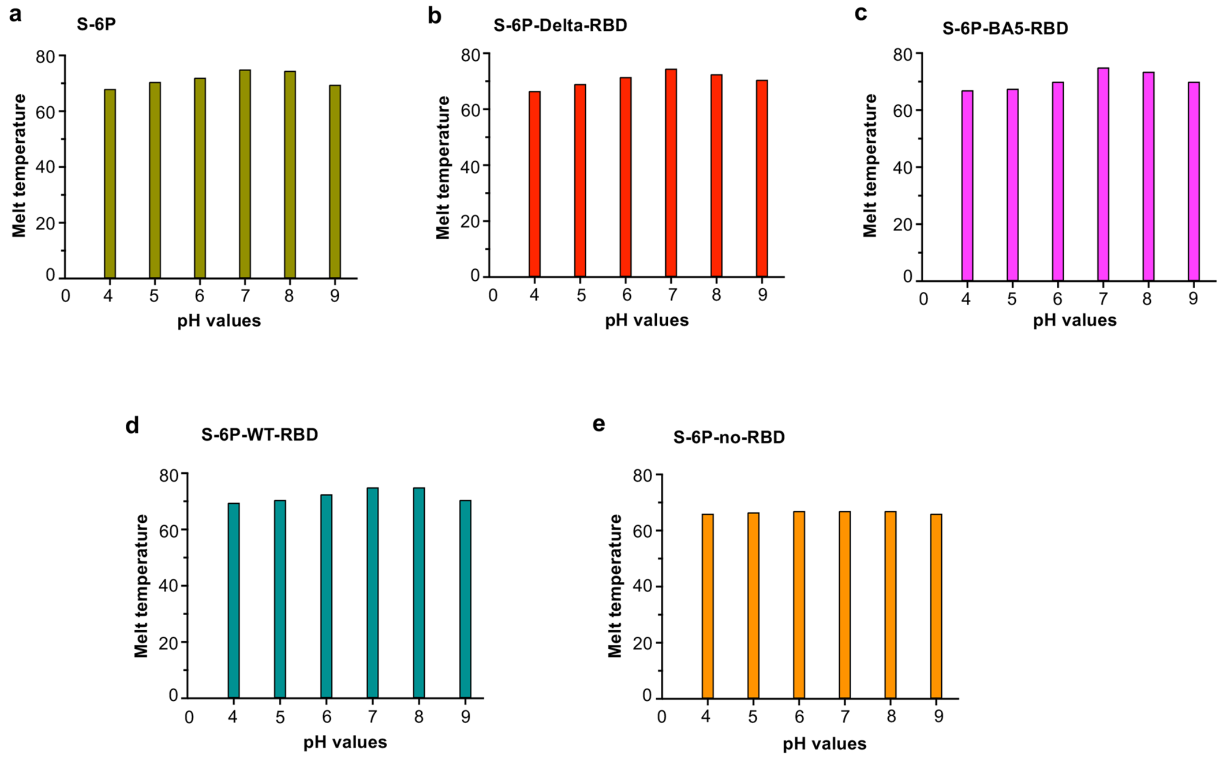

Supplementary Figure 2: Thermal stability of the purified proteins.

Each protein was stained using SYPRO Ruby Protein Gel Stain reagent, and measured for stability using CFX Opus 96 Real-Time PCR System instrument. The data are presented as the Melt Temperature at different temperatures and pH values.

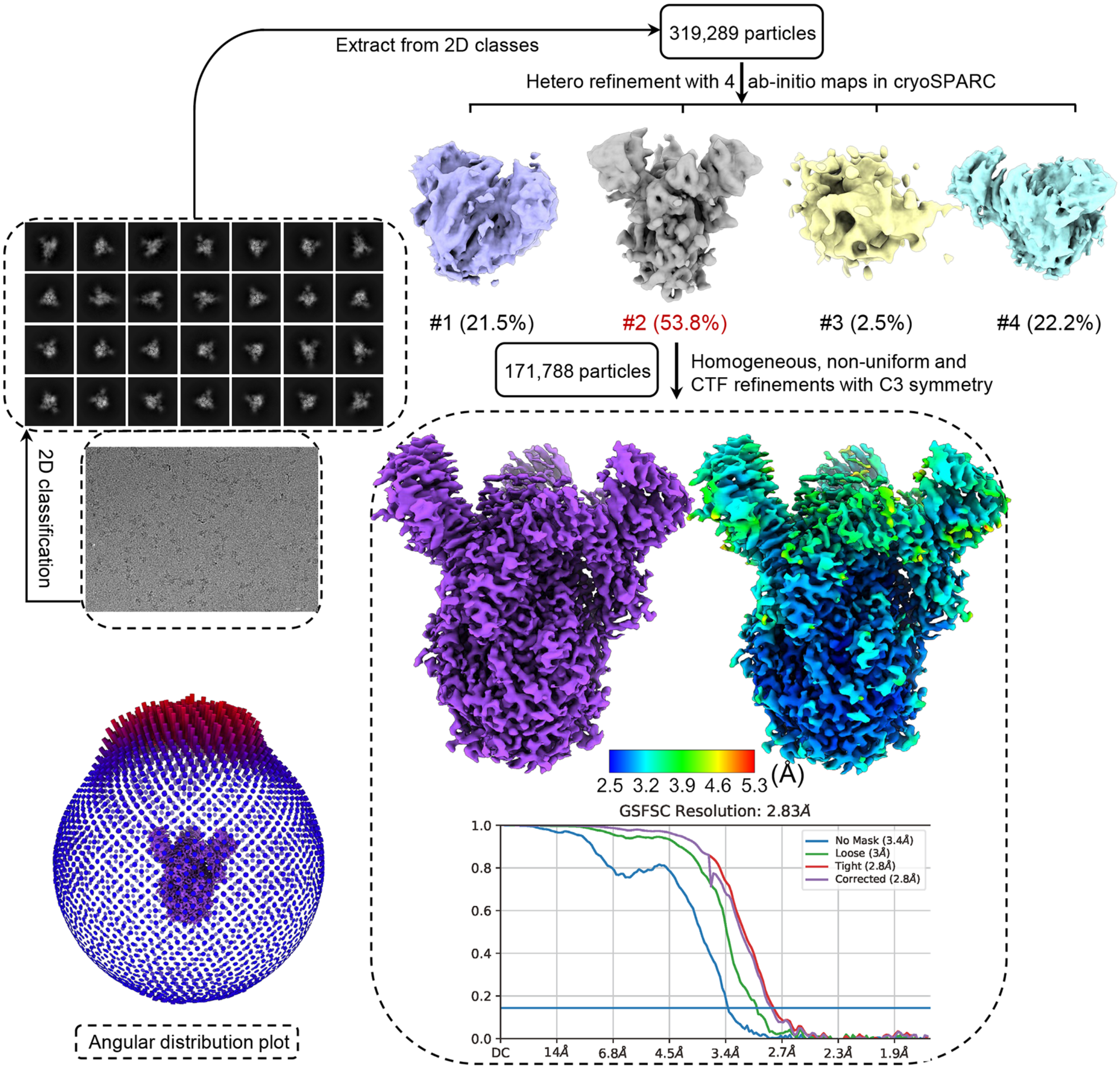

Supplementary Figure 3: Flow chart of cryo-EM data processing for the RBD-truncated S-6P-no-RBD protein.

Representative raw cryo-EM images and 2D classes are presented. Further 3D refinements using the good particles generated an overall 2.8 Å map. Angular distribution plots, the final maps, half-map FSC curves and accompanying local resolution illustrations are displayed.

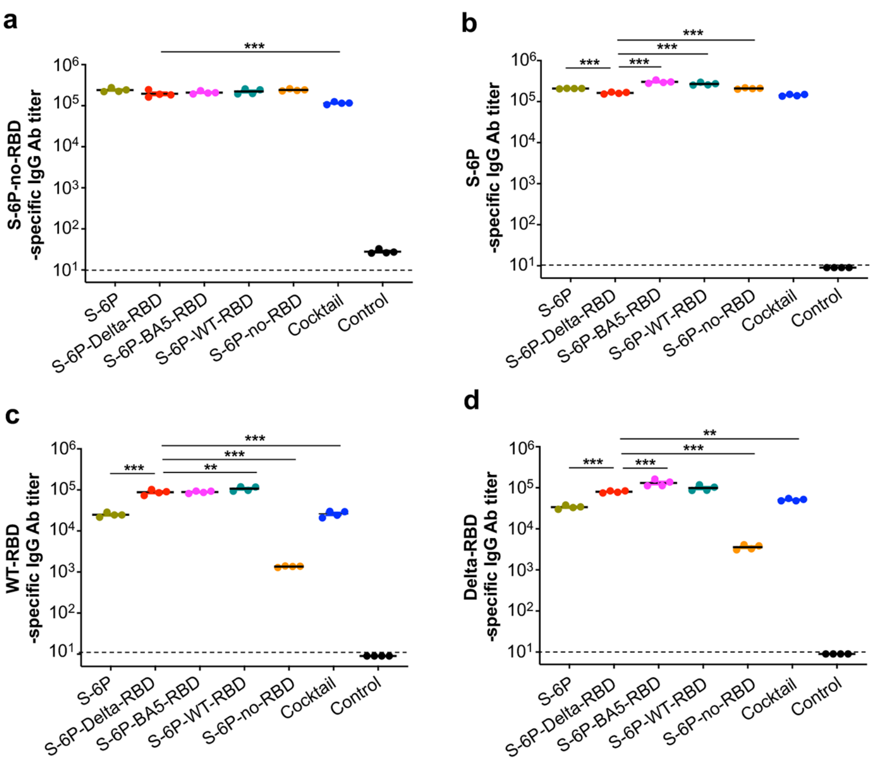

Supplementary Figure 4: The designed subunit vaccines induced SARS-CoV-2-specfiic IgG antibody responses.

K18-hACE2 mice were immunized with each protein, including S-6P, S-6P-Delta-RBD, S-6P-BA5-RBD, S-6P-WT-RBD, and S-6P-no-RBD, the cocktail (combination of S-6P-Delta-RBD and S-6P-BA5-RBD proteins), or PBS control in the presence of adjuvants, and boosted twice at 3 weeks. Sera were collected 10 days after the 3rd immunization, and evaluated by ELISA for IgG antibodies (Abs) specific to the SARS-CoV-2 S-6P-no-RBD (**a**), S-6P (**b**), WT-RBD (**c**), and Delta-RBD (**d**), respectively. The ELISA plates were pre-coated with the respective protein, and the Ab titer was reported as the mean ± standard deviation of the mean (s.e.m.) of four wells from pooled sera of five mice in each group. Ordinary one-way ANOVA (Dunnett's multiple comparison test) was used to compare the statistical differences between S-6P-Delta-RBD and other vaccination groups. Significant differences among different groups are shown as ** ($P <$ 0.01) and *** ($P < 0.001$). The experiments were repeated twice, with similar results.

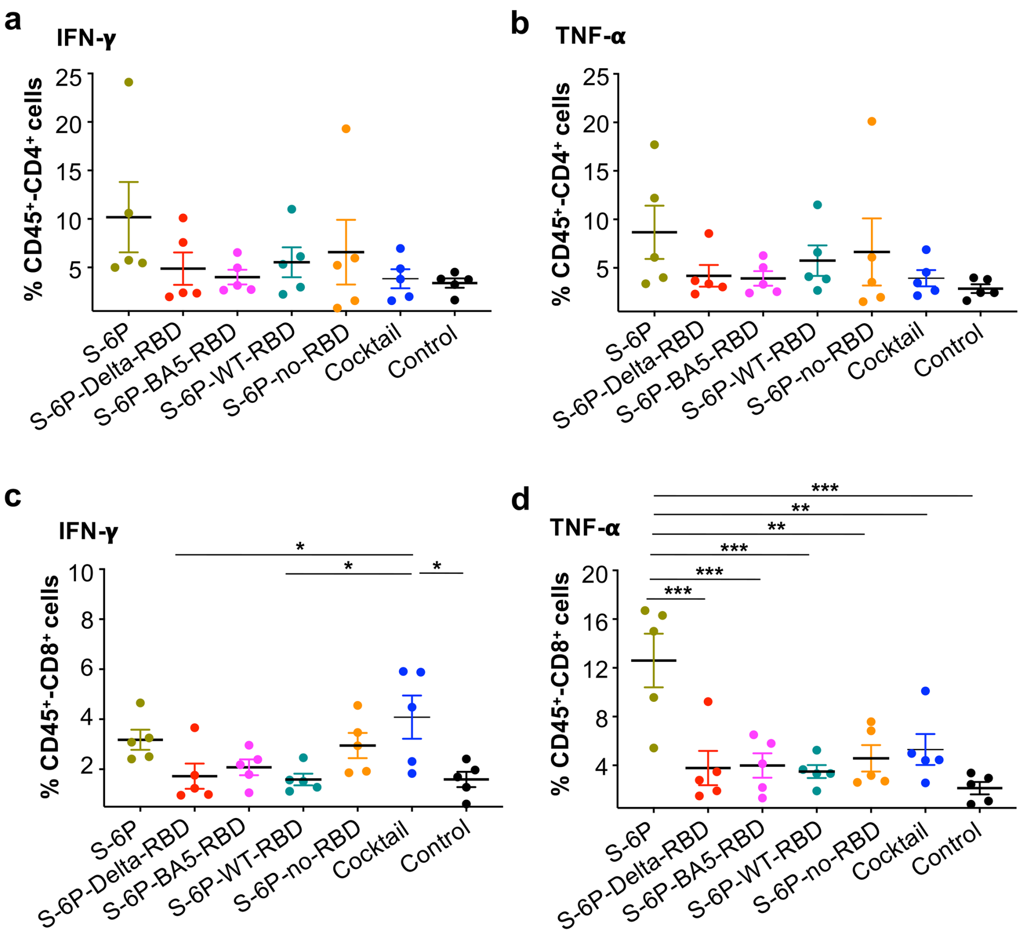

Supplementary Figure 5: The designed subunit vaccines induced SARS-CoV-2-specfiic T cell immune responses.

C57BL/6 mice were immunized with each protein as described in Supplementary Figure 4, and tested for SARS-CoV-2-specific T cell responses in splenocytes 4 months after the 3rd immunization. The splenocytes were stimulated with the S-6P-no-RBD protein, and the IFN-γ or TNF-α-secreting CD4^{+} (**a-b**) and CD8^{+} (**c-d**) T cells were measured by flow cytometry. The data are presented as the mean ± s.e.m of five mice in each group. Ordinary one-way ANOVA (Tukey's multiple comparison test) was used to compare the statistical differences among different groups. * ($P < 0.05$), ** ($P < 0.01$), and *** ($P < 0.001$) designate significant differences among various groups. The experiments were repeated twice, with similar results.

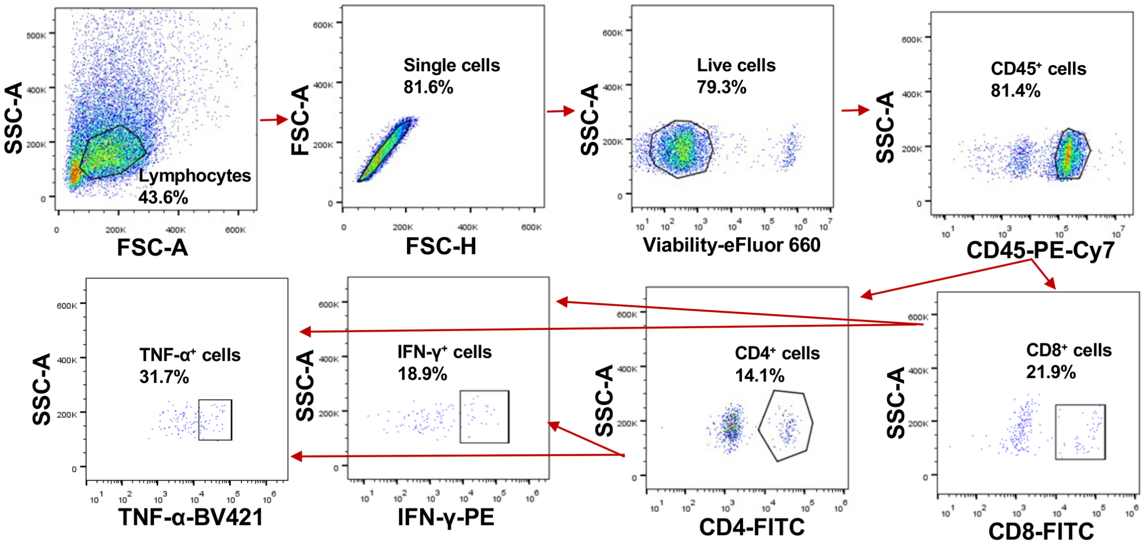

Supplementary Figure 6: Gating strategy for flow cytometry analysis to identify targeted lymphocytes among mouse splenocytes.

The FSC/SSC plot of splenocytes was used to gate lymphocytes based on the size and granularity. Single cells were gated along the diagonal in the FSC-A vs FSC-H plot. Fixable Viability Dye eFluor 660 was applied to include live cells (negative for eFluor 660). Live single cells were then gated for $CD45^+$-$CD4^+$ or $CD45^+$-$CD8^+$ T cells, among which specific cell population for its expression of specialized markers (IFN-γ or TNF-α) was analyzed.

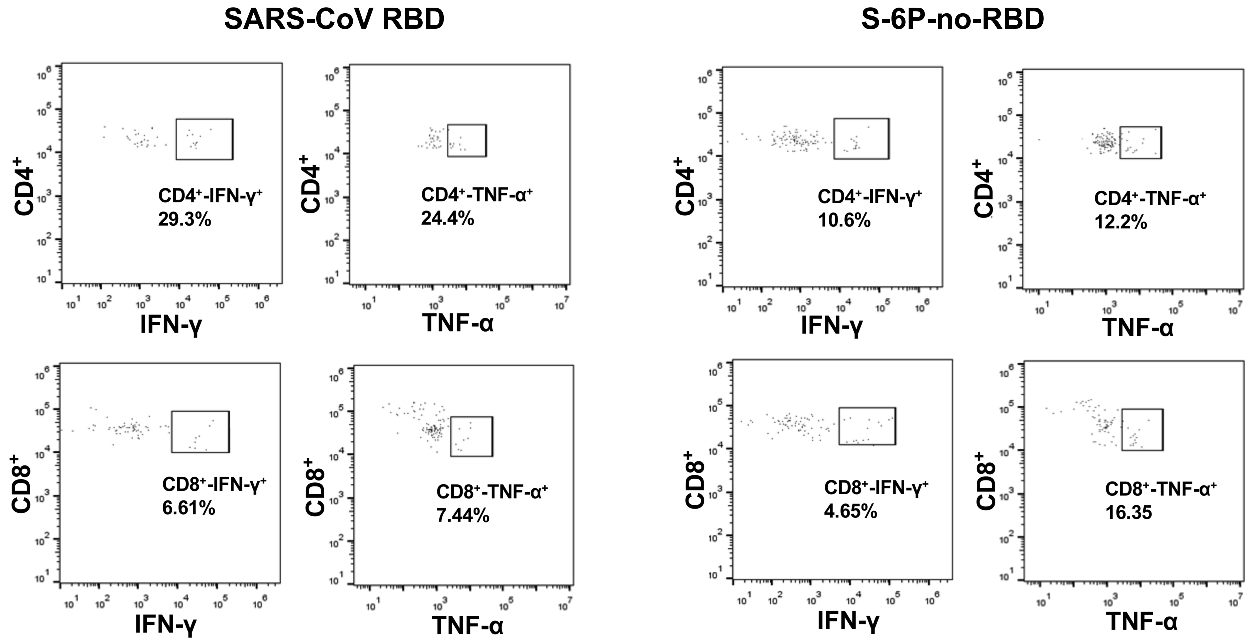

Supplementary Figure 7: Representative flow cytometry plot.

IFN-γ or TNF-α-secreting $CD4^+$ and $CD8^+$ T cells after stimulation with the indicated proteins in the splenocytes of immunized mice are shown.

Supplementary Tables

Supplementary Table 1: Cryo-EM data collection, refinement, and validation statistics.

	S-6P-no-RBD protein (EMDB: EMD-41260; PDB: 8THF)
**Data collection and processing**	
Magnification	130,000
Voltage (kV)	300
Electron exposure (e–/Å^2)	42
Defocus range (μm)	−0.75 ~ −2.5
Pixel size (Å)	0.664
Symmetry imposed	C3
Initial particle images (no.)	319,289
Final particle images (no.)	171,788
Map resolution (Å)	2.8
FSC threshold	0.143
Map resolution range (Å)	2.5-5.3
**Refinement**	
Model resolution (Å)	2.98
FSC threshold	0.5
Map sharpening *B* factor (Å^2)	96.6
Model composition	
Non-hydrogen atoms	21515
Protein residues	2682
Ligands	39
B factors (Å^2)	
Protein	121.52
Ligand	114.04
R.m.s. deviations	
Bond lengths (Å)	1.402
Bond angles (°)	0.011
Validation	
MolProbity score	1.57
Clashscore	3.45
Poor rotamers (%)	0.00
Ramachandran plot	
Favored (%)	93.29
Allowed (%)	6.59
Disallowed (%)	0.11
